# Downregulation of PI3K/AKT/mTOR Pathway in Juglone-Treated Bovine Oocytes

**DOI:** 10.3390/antiox12010114

**Published:** 2023-01-03

**Authors:** Marwa El-Sheikh, Ayman Mesalam, Atif Ali Khan Khalil, Muhammad Idrees, Mi-Jeong Ahn, Ahmed Atef Mesalam, Il-Keun Kong

**Affiliations:** 1 Department of Microbial Biotechnology, Biotechnology Research Institute, National Research Centre (NRC), Dokki, Cairo 12622, Egypt; 2 Department of Theriogenology, Faculty of Veterinary Medicine, Zagazig University, Zagazig 44519, Egypt; 3 Department of Pharmacognosy, Faculty of Pharmaceutical and Allied Health Sciences, Lahore College for Women University, Lahore 54000, Pakistan; 4 Division of Applied Life Science (BK21 Four), Gyeongsang National University, Jinju 52828, Republic of Korea; 5 College of Pharmacy and Research Institute of Pharmaceutical Sciences, Gyeongsang National University, Jinju 52828, Republic of Korea; 6 Department of Therapeutic Chemistry, Pharmaceutical and Drug Industries Research Institute, National Research Centre (NRC), Dokki, Cairo 12622, Egypt; 7 Institute of Agriculture and Life Science, Gyeongsang National University, Jinju 52828, Republic of Korea; 8 The King Kong Corp. Ltd., Gyeongsang National University, Jinju 52828, Republic of Korea

**Keywords:** juglone, oocyte quality, histone methylation, PI3K, AKT, mTOR, signaling

## Abstract

We have previously reported that juglone, a natural compound found in Juglandaceae with a wide range of biological activities, can reduces the developmental competence of bovine oocytes. In the current study, we investigated the possible mechanisms behind the toxicity of juglone and the relationship with PI3K/AKT/mTOR signaling during the in vitro maturation (IVM) of oocytes. Results show that oocyte exposure to juglone was associated with a significant decrease in filamentous actin (F-actin) accumulation. The RT-qPCR showed downregulation of the meiosis progression indicator GSK-3A, oocyte development marker BMP15, mitochondria fusion controlling MFN1, oxidative stress-related OGG1, and histone methylation-related EZH1, EZH2, SUZ12, G9a, and SUV39H2 genes in juglone-treated oocytes. In addition, glycolysis- (PFK1 and GLUT1), ATP synthesis- (ATPase8 and ATP5F1B), and OXPHOS-specific markers (SDHA and SDHD), as well as the oocyte survival regulators (SOD2, VEGF, and MAPK1) significantly decreased upon juglone treatment. Moreover, lower expression of PI3K, AKT, and mTOR was observed at the transcriptional and/or translational level(s). The autophagy markers LC3B and beclin-1 as well as the DNA damage-specific marker 8-OxoG displayed overexpression in juglone-exposed oocytes. Taken together, our results show that administration of juglone during the IVM can reduce the quality and developmental health of bovine oocytes through downregulation of the PI3K/AKT/mTOR pathway and its downstream signaling cascades.

## 1. Introduction

The capability of oocytes to develop embryos depends mainly on the quality of oocyte that can be affected by different factors, including the conditions of in vitro maturation (IVM) [1]. The synthesis of mRNA and protein during the development and maturation of oocyte is also linked to the quality of oocytes to generate embryos [1]. On the other hand, histone modification is a key post-translational epigenetic regulator that has a vital role in controlling gene expression, and thereby affecting the different cellular processes. In oocyte, histone alternation is an important hallmark involved in the control of oocyte quality and development. Disturbance in histone methyltransferases causes a delay in oocyte maturation, and affects several meiotic progression events, such as chromosome segregation and organization, and finally contributes to oocyte aging [2].

During oocyte maturation, the supplementation of IVM medium with different anti-developmental agents and specific inhibitors for critical pathways regulating cell survival and function, such as the PI3K/AKT pathway, can attenuate the quality of oocyte, disrupt histone modification and mitochondrial activity, promote apoptosis, and alter the expression of different genes related to DNA damage, proliferation, cell cycle, mitochondrial fission/fusion, and histone methylation [3,4,5]. For example, in vitro treatment of bovine oocytes with the SH6 (specific AKT inhibitor) significantly downregulated the expression of AKT in oocytes [4].

Juglone, 5-hydroxy-1,4-naphthalenedione, is a natural compound found in walnut trees (Juglans). It exhibits wide biological activities, including anticancer characteristics documented in hepatocellular carcinoma (HCC), leukemia, melanoma, gastric, and prostate cancer [6,7,8,9,10]. The effect of juglone on cancer cells occurred via different mechanisms, including the induction of apoptosis, autophagy, inhibition of cell proliferation, invasion and migration of cells, in addition to its influence on different cell signaling pathways, such as interleukin 6 (IL-6)/STAT3, AMP-activated protein kinase (AMPK), mitogen- activated protein kinases (MAPK), glycogen synthase kinase-3 (GSK3) protein kinases, and the PI3K/AKT/mTOR [6,7,8,9,10,11]. Despite the cytotoxicity of juglone, it was used in treatment of some conditions such as abdominal disorders, in addition to its anti-microbial, and anti-allergic effects [12].

The AKT, or protein kinase B (AKT/PKB), is a serine (Ser)/threonine (Thr) protein kinase that is a key mediator of the phosphatidylinositol 3-kinase (PI3K) [13]. Mammalian target of rapamycin (mTOR) is a downstream product of AKT and the full functionality of AKT requires the phosphorylation of AKT (at Thr308 and Ser473 phosphorylation sites), the later which mediates the activation of mTOR complex 2 [14,15]. The cooperation between AKT with its upstream PI3K and downstream mTOR, as well as with other protein kinases, can influence the quality and development of oocytes [16,17].

Previously, we identified a deleterious effect of juglone, administered during the IVM step, on the developmental competence of preimplantation embryos by induction of apoptosis in oocyte [18]. In the current study using bovine oocyte as a model, we sought to explore the quality of oocytes as well as the PI3K/AKT/mTOR signaling pathway post juglone treatment. The different markers related to cell survival, histone methylation, autophagy, aerobic glycolysis, ATP synthesis, and oxidative phosphorylation reactions were also studied at transcriptional and/or translational levels.

## 2. Materials and Methods

### 2.1. Oocytes Collection, In Vitro Maturation (IVM), and Juglone Treatment

Ovaries from Hanwoo cows were collected at a local abattoir, transported to the laboratory within 2 h after slaughter, and washed in fresh Dulbecco’s phosphate-buffered saline (D-PBS). Follicles with a diameter of 2–8 mm were aspirated using 18-gauge needles attached to a vacuum pumps. Cumulus-oocyte complexes (COCs) with compact cumulus cells were picked up and washed three times in IVM medium (TCM-199 supplemented with 10% FBS, 1 µg/mL estradiol-17ß, 10 µg/mL follicle-stimulating hormone, 10 ng/mL EGF, 0.6 mM cysteine, and 0.2 mM sodium pyruvate).

Previously, we explored the effect of different juglone concentrations (12.5, 25, and 50 µM) on oxidative stress in bovine oocyte and the developmental competence of embryos [18]. Based on our earlier data, juglone at 25 µM concentration significantly reduced the cleavage and blastocyst production rates [18]. In the present study, we used 25 µM of juglone to check its impact on the quality and developmental health of oocytes. Groups of around 50 COCs were cultured in four-well dishes containing 500 µL of IVM medium (in presence or absence of 25 µM of juglone) and incubated at 38.5 °C and 5% CO_2_ for 22–24 h. The experimental procedures were approved by the Institutional Animal Care and Use Committee of the Division of Applied Life Sciences (Approval ID: GAR-110502-X0017). The reagents used in the study were obtained from Sigma-Aldrich (St. Louis, MO, USA) unless otherwise stated.

### 2.2. Visualization of Cytoskeleton

Oocytes (Four replicates; n = 20) were denuded by repeated pipetting and fixed in 4% paraformaldehyde followed by co-incubation with blocking buffer (10% donkey serum/3% BSA in PBS) for 2 h. Oocytes were then stained with Alexa Fluor 488–phalloidin for 3 h, washed three times and mounted on glass slides with Prolong anti-fade reagent. Stained oocytes were examined using a confocal laser-scanning Olympus Fluoview FV1000 microscope (Olympus, Tokyo, Japan) and the optical densities were estimated using ImageJ.

### 2.3. RNA Extraction and RT-qPCR

Total RNA was extracted from oocytes (n = 50, triplicate) using the Arcturus PicoPure RNA isolation kit according to the manufacturer’s guidelines (Arcturus, Foster, CA, USA). The concentration of RNA was estimated using NanoDrop 2000c spectrophotometer (Thermo Fisher Scientific, Waltham, MA, USA) and the cDNA was synthesized using an iScript cDNA synthesis kit (Bio-Rad Laboratories, Hercules, CA, USA). The RT-qPCR was carried out using iQ-SYBR Green Supermix (Bio-Rad Laboratories) according to the manufacturer’s instructions and the real-time PCR amplification was carried out on CFX96 instrument (Bio-Rad Laboratories). The sequences of primers are listed in Table S1. The relative gene expression is presented as fold change compared to the control group following the 2^−ΔΔCT^ method.

### 2.4. Immunofluorescence

Denuded oocytes (Four replicates; n = 20), fixed in 4% paraformaldehyde, were permeabilized using 0.5% Triton X-100 and left in blocking buffer (10% donkey serum/3% BSA) for 4 h at room temperature before incubation overnight at 4 °C with the primary antibodies (Table S2). Oocytes were washed three times and incubated for 90 min at room temperature with the secondary antibodies (Table S2), and then incubated with Hoechst 33342 (10 mg/mL) for 15 min. Samples were mounted on glass slides with Prolong anti-fade reagent and examined under confocal laser-scanning Olympus microscope while the optical densities were estimated using ImageJ.

### 2.5. Statistical Analysis

Data were analyzed using GraphPad Prism version 6 (San Diego, CA, USA) and the differences between groups were analyzed using Student’s *t*-test. All values are presented as the mean ± standard error of the mean while the *p*-values below 0.05 were considered statistically significant.

## 3. Results

### 3.1. Juglone Addministration Affects the Quality of Oocytes

**Figure 1:**
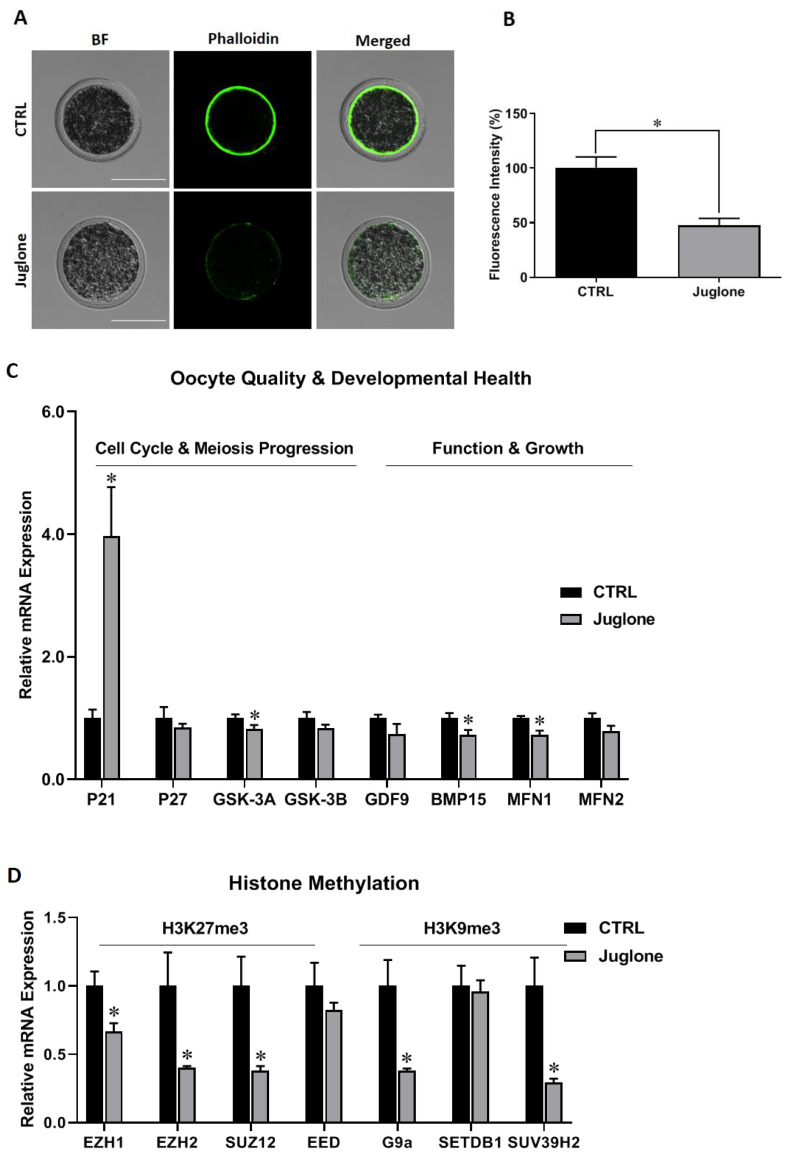
The effect of juglone treatment on the quality and developmental health of bovine oocytes. Oocytes were treated with 25 µM of juglone during in vitro maturation (IVM), four replicates (n = 20), and three replicates (n = 50) were used for determining the filamentous actin (F-actin) and RT-qPCR experiments, respectively. (**A**) Phalloidin-based staining explored the distribution of F-actin in oocytes. (**B**) Fluorescence intensity of stained F-actin in treated and untreated oocytes. (**C**,**D**) Relative expression of oocyte quality- and development- related genes. (**C**) The mRNA levels of P21, P27/GSK-3A, GSK-3B/ GDF9, BMP15/MFN1, MFN2 regulating cell cycle, meiosis progression, oocyte function and mitochondrial fusion, respectively. (**D**) The mRNA levels of histone methylation H3K27me3 (EZH1, EZH2, SUZ12, and EED), and H3K9me3 (G9a, SETDB1 and SUV39H2)-related methyltransferases. Scale bar = 100 μm. Columns with an asterisk (*) indicate statistical significance. BF: Bright field.

We start to check the quality of bovine oocytes upon exposure to juglone. Using the phalloidin-based staining, the fluorescence intensity corresponding to filamentous actin (F-actin) significantly decreased in matured oocytes after juglone exposure compared to the untreated control (Figure 1A,B).

Additionally, we checked the mRNA expression of oocyte quality and development markers. As seen in Figure 1C, P21 was significantly upregulated whereas as GSK-3A and BMP15 were downregulated post juglone treatment. Additionally, oocyte quality- mitochondrial fusion markers (MFN1 and MFN2) were investigated. As shown in Figure 1C, a significant reduction in the transcription of MFN1 was witnessed in juglone-treated oocytes compared to untreated ones. Next, testing the mRNA levels of the histone methylation, a critical process for oocyte development, -specific genes revealed downregulation of EZH1, EZH2, SUZ12, G9a, and SUV39H2 after juglone treatment (Figure 1D).

### 3.2. Modulation of PI3K/AKT/mTOR Signaling in Oocytes

**Figure 2:**
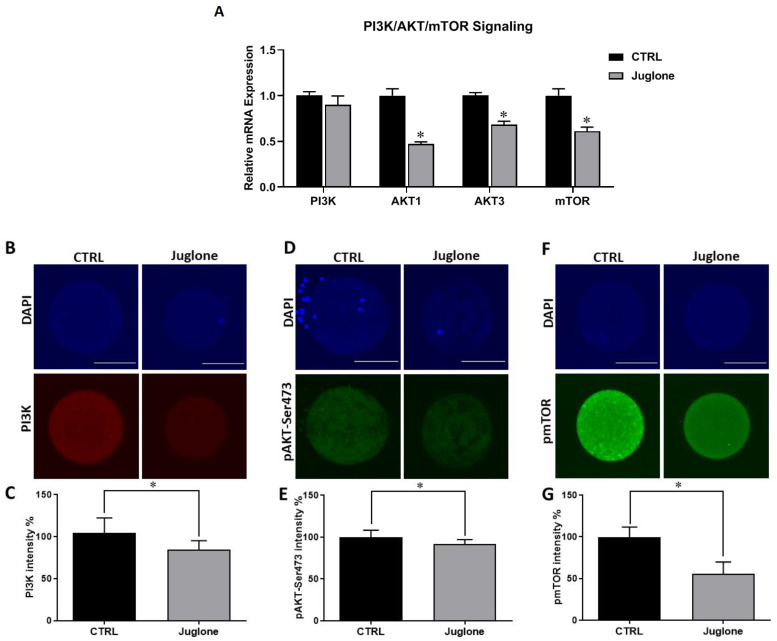
The effect of juglone on the PI3K/AKT/mTOR pathway in oocytes. Bovine oocytes were exposed to 25 µM of juglone during in vitro maturation (IVM), three replicate (n = 50), and four replicates (n = 20) were used for RT-qPCR and immunofluorescence analysis, respectively. (**A**) Relative expression of PI3K, AKT, and mTOR genes using RT-qPCR. (**B**,**D**,**F**) Immunofluorescence of PI3K, pAKT, and pmTOR in juglone treated and untreated oocytes. (**C**,**E**,**G**) Fluorescence intensities of PI3K, pAKT-Ser473, and pmTOR in juglone-treated and control groups. Scale bar = 100 μm. Columns with an asterisk (*) indicate statistical significance. DAPI: 4′,6-diamidino-2-phenylindole. pAKT: phosphorylated AKT; and pmTOR: phosphorylated mTOR.

To clarify the possible mechanisms behind the deleterious effect of juglone on oocyte quality, we sought to investigate the PI3K/AKT/mTOR signaling pathway, a critical parameter controlling oocyte development and survival, at transcription and translation levels. The use of RT-qPCR revealed significant downregulation of AKT1, AKT3, and mTOR genes in juglone-treated oocytes (Figure 2A). Similarly, immunofluorescence analysis showed a dramatic decrease in expression of PI3K, pAKT, and pmTOR proteins in oocytes of the treated group compared to the control (Figure 2B–G).

### 3.3. Downstream Cascades Affected by Juglone

PI3k/AKT/mTOR signaling regulates different cellular processes, including autophagy, DNA damage, and different metabolic pathways. We hypothesized that the downregulation of PI3k/AKT/mTOR in oocytes, following juglone exposure, might induce cellular damage through disturbing the critical processes in the downstream pathway. Hence, we moved forward to investigate the effect of juglone on autophagy, DNA damage, glucose metabolism, ATP synthesis, and oxidative phosphorylation (OXPHOS) reactions in oocyte.

#### 3.3.1. Induction of Autophagy

**Figure 3:**
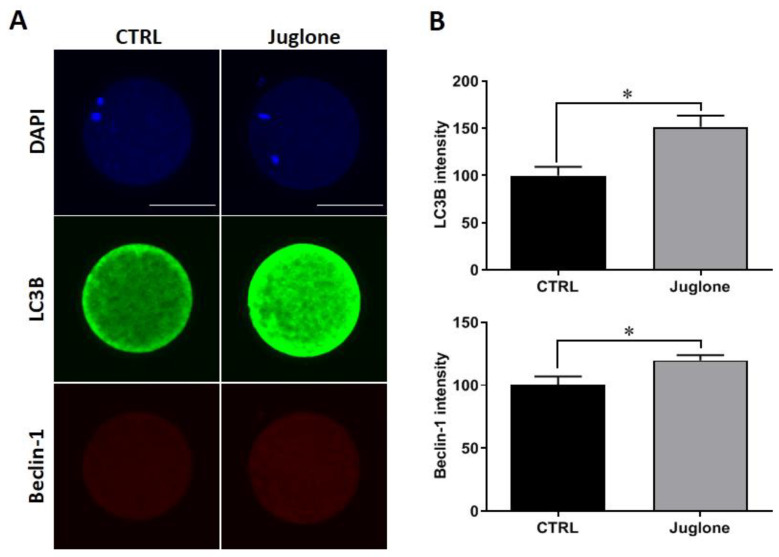
Effect of juglone supplementation during IVM on autophagy. Juglone was treated at 25 µM during in vitro maturation (IVM) of bovine oocytes, four replicates (n = 20) were used for immunofluorescence analysis. (**A**) Immunofluorescence of Beclin-1 and LC3B in treated and untreated oocytes. (**B**) Fluorescence intensities of Beclin-1 and LC3B. Scale bar = 100 μm. Columns with an asterisk (*) indicate statistical significance. DAPI: 4′,6-diamidino-2-phenylindole; LC3B (MAP1LC3B): microtubule-associated protein 1 light chain 3 beta; and Beclin-1: autophagy-related gene 6.

Checking the protein expression of the autophagy-related markers demonstrated significant increase in the levels of LC3B and beclin-1 in juglone-exposed oocytes compared to untreated ones (Figure 3A,B).

#### 3.3.2. Induction of DNA Damage

**Figure 4:**
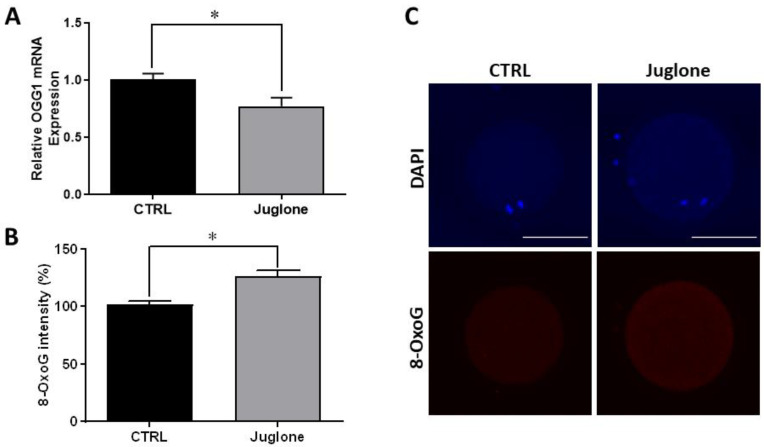
Effect of juglone (25 µM) supplementation during IVM of bovine oocytes on DNA damage, four replicates (n = 20) were used for immunofluorescence analysis. (**A**) Relative gene expression of OGG1. (**B**) Fluorescence intensities of 8-OxoG expression level. (**C**) Images representing the immunofluorescence of 8-OxoG in juglone-treated and the untreated oocytes. Scale bar = 100 μm. Columns with asterisk (*) indicate statistical significance. OGG1: 8-Oxoguanine DNA Glycosylase-1; 8-OxoG: 8-Oxoguanine; and DAPI: 4′,6-diamidino-2-phenylindole.

Since the PI3K/AKT/mTOR signaling plays a critical role in the control of the DNA repair mechanisms in several cell lines, we further executed to check the effect of juglone on DNA damage using the DNA damage-specific marker (8-Oxoguanine; 8-OxoG). In addition, the mRNA expression of 8-OxoG DNA Glycosylase-1 (OGG1), the gene responsible for 8-OxoG excision, was also inspected. As seen in Figure 4, the results of RT-qPCR and immunofluorescence reveal a significant decrease in the transcription pattern of OGG1 and overexpression of the 8-OxoG protein level, respectively, in treated oocytes.

#### 3.3.3. Downregulation of Aerobic Glycolysis, ATP Synthesis, and Oxidative Phosphorylation Reactions

**Figure 5:**
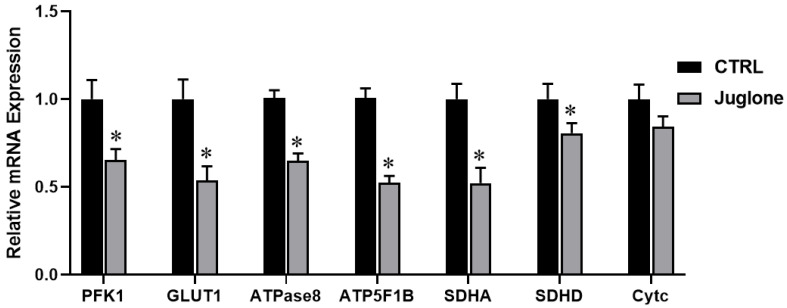
The relative expression of metabolic pathways in oocytes using glucose metabolism-, ATP synthesis-, and OXPHOS-related genes tested using RT-qPCR (three replicate; n = 50). Bovine oocytes were incubated with 25 µM of juglone during in vitro maturation (IVM). Columns with an asterisk (*) indicate statistical significance.

Moving forward, we checked the transcriptional levels of glucose metabolism, ATP synthesis, and mitochondrial OXPHOS reactions in oocytes after administration of juglone. As seen in Figure 5, the glycolysis-specific genes (PFK1 and GLUT1) were significantly downregulated in juglone-treated oocytes when compared to untreated control. Moreover, the RT-qPCR results of the ATP synthesis (ATPase8, ATP5F1B) genes show significant decrease of in both genes oocytes of the treated group (Figure 5). Likely, SDHA and SDHD (the markers for OXPHOS) significantly decreased albeit the non-significant downregulation of cytochrome c (Cytc; the component of electron transport chain in mitochondria) (Figure 5).

#### 3.3.4. Reduction in Oocyte Survival Rate following Juglone Exposure

**Figure 6:**
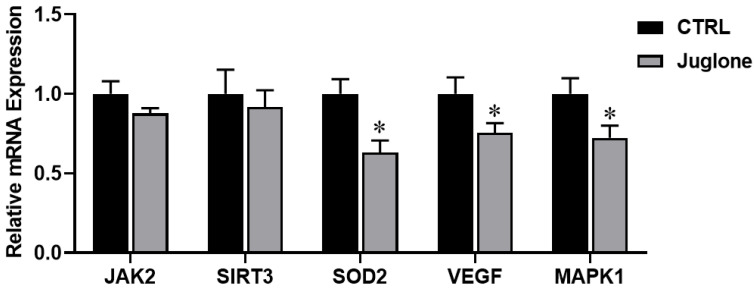
The relative expression of oocyte survival-related genes tested using RT-qPCR. Bovine oocytes were treated with 25 µM of juglone during in vitro maturation (IVM) and three replicate; n = 50 were used for the experiment. Columns with asterisk (*) indicate statistical significance.

Finally, we demonstrated the impact of juglone treatment on the modulation of genes critical for oocyte survival (such as JAK2, SIRT3, SOD2, VEGF, and MAPK1). As seen in Figure 6, significant reduction in SOD2, VEGF, and MAPK1 genes was observed in the treated group as compared to control.

**Figure 7:**
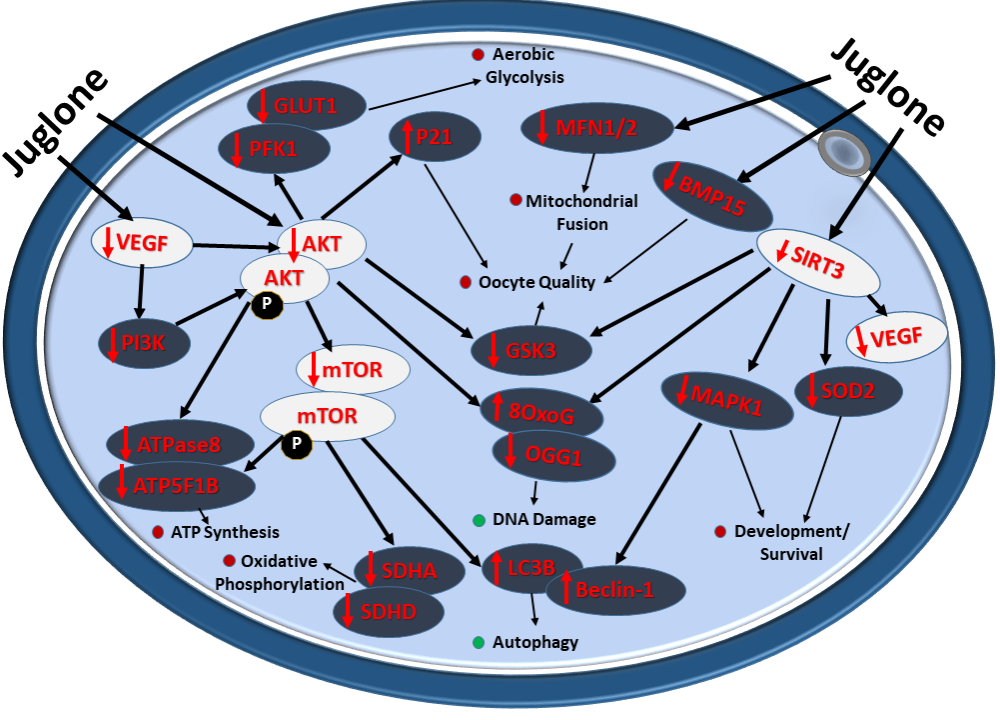
Diagram showing the deleterious impact of juglone implication on bovine oocyte during IVM. Juglone interact with the PI3K/AKT/mTOR signaling and the underlying cascades regulated by this pathway, the effects that negatively retard the development and quality of exposed oocyte. Red and green dots indicate the decrease and increase in the cellular processes affected by juglone in bovine matured oocyte, respectively.

The overall impact of juglone administration during IVM on the quality and developmental health of bovine oocytes is summarized in Figure 7.

## 4. Discussion

Manipulation of gametes during the in vitro production process can alter gene/protein expression and phenotypes in pre- and post-implantation embryos [19]. Juglone, a natural naphthoquinone, is used in traditional Chinese medicine [7,11]. The cytotoxicity of juglone through the induction of apoptosis as well as inhibiting mRNA and protein synthesis was reported [8,11,20,21]. Despite the multiple pharmacological actions of juglone, very few studies have investigated its impact on embryonic development [18,20], whereas little is known about the molecular mechanism behind the effects of juglone on female reproduction, particularly oocyte development. In the current study, we used bovine oocyte as a model to study the effect of juglone, administered during the IVM, on the developmental quality of oocytes.

Actin is a multifunctional protein playing crucial roles during oogenesis, fertilization, and embryo development [22]. The abnormal abundance and distribution of filamentous actin observed in oocytes after juglone treatment match with previous findings on its anti-developmental effect in bovine and porcine oocytes [18,20]. Next, we studied the effect of juglone on the levels of mRNA markers involved in the regulation of oocyte quality and development (including the cell cycle and meiosis progression (P21, P27, GSK-3A, and GSK-3B) and oocyte functionality (GDF9, BMP15). The RT-qPCR results show upregulation of P21 and downregulation of GSK-3A, BMP15, and MFN1 genes following juglone treatment, confirming the toxic effect of juglone. These observations are in line with the previous studies that showed upregulated P21 expression in response to stress in various cell types [23]. The two oocyte secretion factors (GDF9 and BMP15) and the mitochondrial fusion markers (MFN1 and MFN2) are critical for oocyte development and quality [24,25,26,27]. The MFN1 and MFN2 control mitochondrial dynamics by altering mitochondrial health, membrane potential, and distribution pattern. They are also regulatory parameters for apoptosis, cellular metabolism, and aerobic respiration [27,28]. Importantly, it was reported that the deletion of MFN1 was associated with defects in oocyte development and impairment of PI3K/AKT signaling [26].

Histone modification is another critical parameter that affects the quality and development of oocytes and embryos [2]. In the current study, a downregulation of H3K9me3 and H3K27me3 methyltransferases (EZH1, EZH2, SUZ12, G9a, and SUV39H2) were observed in oocytes after juglone treatment. Previously, treatment of oocytes with paraquat herbicide downregulated the expression of both H3K9me2 and H3K27me3 in mouse oocyte [29]. Additionally, a significant decrease in the level of H3K9me2 was observed after exposing bovine oocytes to lipopolysaccharide toxin [30]. Although these findings are contradicted by Han et al., who reported increased H3K9me3 (G9a, SUV39H2) and H3K27me3 (EZH1, EZH2, and SUZ12) methyltransferases mRNA levels in oocyte exposed to the mycotoxin deoxynivalenol [31], the results confirm the direct effect of juglone on the alternation of histone methylation in matured oocyte. It was reported that the PI3K/AKT pathway plays an important role in regulating the epigenome to promote oncogenesis as AKT-mediated phosphorylation of the histone methyltransferase EZH2 decreases EZH2 affinity for chromatin, reducing the repressive promoter-associated H3K27me3 modification. Phosphorylation of the histone demethylase KDM5A increases its cytoplasmic localization, thereby increasing promoter H3K4me3 and transcriptional competence [32].

Based on accumulating evidence suggesting a linkage between PI3K/AKT/mTOR signaling and oocyte maturation and ovarian function [4,17,33], we sought to investigate whether this pathway is implicated in the anti-developmental effect of juglone. Interestingly, downregulation of PI3K, AKT, and mTOR was witnessed in juglone-treated oocytes. Previously, it was shown that AKT is responsible for the completion of meiosis, while the specific inhibition of AKT resulted in meiotic arrest at the MI stage and hindered the development of embryos [4,34,35]. It was reported that juglone inhibited PI3K activity in mouse skin epidermal cells in vitro by directly binding with PI3K [36]. Additionally, inhibitors for PI3K/AKT signaling adversely affected the developmental competences of oocytes and embryos in vitro [3,4]. The mTOR was reported to be expressed in all stages of oocytes development, suggesting its vital role in oocytes meiosis completion and further embryonic development [37]. Interestingly, activation of mTOR in cumulus cells must be ensured in order to produce a functionally normal eggs [38]. This matches with our study that demonstrated inhibition of PI3K/AKT/mTOR signaling pathway components following juglone treatment could be attributed to inferior oocyte quality.

Then, we further investigate the cascades, downstream to PI3K/AKT/mTOR, that are related to different cellular processes (including autophagy, DNA repair, aerobic glycolysis, ATP synthesis, and OXPHOS reactions), as well as the levels of the oocyte survival related markers in juglone-treated oocytes. Based on our results, the levels of protein expression of the autophagy markers LC3B and beclin-1 showed significant increase in juglone-exposed oocytes, confirming the detrimental effect of juglone on oocyte developmental competence. In line with this, hyper-activated autophagy during oocyte maturation following exposure of oocyte to toxic compounds was reported to negatively affect the oocyte and embryonic development competences [33,39]. Additionally, the toxicity of juglone reported in multiple studies was mainly attributed to its ability to induce autophagy, DNA damage, endogenous reactive oxygen species (ROS) accumulation, apoptosis, and meiotic defect in oocyte [6,7,20,21]. Additionally, the enhancement in the rate of autophagic cell death in human HL-60 promyelocytic leukemia cells, as well as in the expressions of LC3-II and Beclin-1, was reported in a dose-dependent manner [7]. In line with these previously mentioned results, we detected an overexpression of 8-OxoG protein levels and downregulation of OGG1 expression in juglone-treated oocytes. The common DNA oxidation product, 8-OxoG, is produced when DNA is exposed to ROS. Likewise, juglone treatment was associated with increased ROS levels in oocytes in a dose-dependent manner [18]. During stress conditions, OGG1 stimulates the removal of 8-oxoG through glycosylase and endonuclease activities, which indicates the protection against the oxidative stress [40].

Moreover, the different metabolic pathway markers were checked. These regulatory parameters for aerobic glycolysis, ATP synthesis, and OXPHOS reactions were investigated in juglone-treated oocytes. We detected a strong downregulation in the glycolysis PFK1 and GLUT1, the ATP synthesis ATPase8 and ATP5F1B, and OXPHOS SDHA and SDHD genes in juglone-exposed oocytes. Recently, a decrease in mRNA and protein levels of PFK1 and GLUT1 caused inhibition of aerobic glycolysis in human umbilical vein endothelial cells [41]. Importantly, AKT and mTOR are key regulators for metabolic flux and glycolysis via controlling the activity of PFK1 and GLUT1, which confirms our abovementioned data for the decrease in the PI3K/AKT/mTOR in juglone-treated oocytes.

We moved forward to assess the levels of oocyte survival related genes, we demonstrated the suppression of the expression SOD2, VEGF, and MAPK1 genes in juglone treated oocytes. These data suggest that there is a possible linkage between juglone and reduced oocyte quality. This supports the previous study that showed antioxidant enzyme SOD2 plays a crucial role in controlling ROS production and poor quality of oocytes from diabetic mice that lack the capacity to promote SOD2 activity in response to oxidative stress [42]. Vascular endothelial growth factor (VEGF), which is involved in oocyte maturation and embryo development [43], was reported to improve the revascularization, survival, and oocyte quality of cryopreserved, subcutaneously-transplanted mouse ovarian tissue [44]. Mitogen-activated protein kinase (MAPK) is involved in regulating preimplantation embryo development [45]. Surprisingly, the suppression of MAPK activation in porcine oocytes decreased the maturation promoting factor activity [46], while in mouse embryos, it resulted in a reversible developmental blockade at the 8–16 cell stage [45]. Interestingly, a direct linkage between MAPK signaling and glucose metabolism was previously reported, which supports our data for the decrease in the glycolysis markers in juglone oocyte [47].

## 5. Conclusions

Collectively, we report that juglone can significantly affect the development of bovine oocytes through the direct induction of autophagy, as well as DNA and mitochondrial damage. Additionally, the negative effects of juglone on oocyte quality, histone methylation, glucose metabolism, ATP synthesis, and oxidative phosphorylation reactions were observed. Finally, to our knowledge, this is the first study reporting that juglone, administered during IVM, can significantly interact with the PI3K/AKT/mTOR signaling pathway and its downstream signaling cascades in bovine oocytes.

## Data Availability

All of the data is contained within the article and the supplementary materials.

## Supplementary Materials

The following supporting information can be downloaded at: https://www.mdpi.com/article/10.3390/antiox12010114/s1, Table S1: The names of the genes and sequences of the primer used in RT-qPCR analysis; Table S2: List of Antibodies used for immunofluorescence analysis.
