# Supplementary material for: Downregulation of PI3K/AKT/mTOR Pathway in Juglone-Treated Bovine Oocytes
Source: Antioxidants (Basel). 2023 Jan 3;12(1):114. doi: 10.3390/antiox12010114 (PMC9854430; doi:10.3390/antiox12010114)
Supplement: Supplementary file 1 [file antioxidants-12-00114-s001.zip › antioxidants-2102267-supplementary.pdf]

**Table S1:** The names of the genes and sequences of the primer used in RT-qPCR analysis.

| Gene name | Accession no. | Amplicon (bp) | Forward Primer | Reverse Primer |
|---|---|---|---|---|
| P21 | NM_001098958.2 | 112 | GCAAATATGGGTCTGGGAGA | AAATAGTCCAGGCCAGGATG |
| P27 | XM_019961532.1 | 150 | TGTCAAACGTGCGAGTGTCTA | CTCTGCAGTGCTTCTCCAAGT |
| GSK-3A | XM_024978404.1 | 105 | GGCTCATTTGGGGTCGTGTA | CTGCAGCTCTCGGTTCTTGA |
| GSK-3B | XM_027529592.1 | 161 | CCTTCCTTCCTTCCCCACAC | GCTGCCAAGAGACTCCACTT |
| GDF9 | NM_174681.2 | 123 | CAGAAGCCACCTCTACAACACTG | CTGATGGAAGGGTTCCTGCTG |
| BMP15 | NM_001031752.1 | 141 | GAGGCTCCTGGCACATACAGAC | CTCCACATGGCAGGAGAGGT |
| MFN1 | NM_001206508.1 | 93 | CCCTACAAATGCTCAGCGGG | TTGGAAGGAGCAGTGGGAGT |
| MFN2 | XM_024976324.1 | 156 | AAGATGGCGGAAGATGGGGA | TAAGCTGGTAGGGTGGGCTC |
| EZH1 | XM_015458835.2 | 144 | ATCTTCCGGGTAGTGGGGTG | ATGGAACCTGGGAGCTCCTC |
| EZH2 | XM_024990587.1 | 168 | GCAACGACAAGGACGAGGAG | AGTAGGTGCCGATGAGGACC |
| SUZ12 | NM_001205587.3 | 178 | GATATTCATCGCCAGCCCGG | GGCGGTTGTGTCCACTACTG |
| EED | XM_015461113.2 | 113 | TAGCAACCCGGACCTCTCTG | TCCTTCCAGGTGCATTTGGC |
| G9A | XM_024983572.1 | 131 | GATTTCCACGCATCGCCTTC | GCACTTCTCAGAGCCACACT |
| SETDB1 | NM_001191388.3 | 101 | TCTATCGAGGCTCTACGCGA | TGGACGTGTTCTGAGCTGTC |
| SUV39H2 | XM_005214231.4 | 100 | GTGTCCAACGTGACAGCAAC | AGTGCTGCATCCCTGAAGTC |
| PI3K | NM_174574.1 234 | 234 | TCAACCATGACTGTGTGCCA | CCATCAGCATCAAATTGGGCA |
| AKT1 | NM_173986.2 | 80 | AAAAGGAAGTGGTGTACAGG | GAAGTCGGTGATCTTGATGT |
| AKT3 | NM_001191309.1 | 94 | AGCTGTTTTTCCATTTGTCG | TGTAGATAGTCCAAGGCAGA |
| mTOR | XM_002694043.6 | 113 | TTAACAGGGTTCGAGAGAAG | AGAGGTTTTCATGGGATGTC |
| OGG1 | XM_010817459.3 | 140 | CAACTCCAGCAAGCTCAGGA | GAGACTGGATGGGGGAGAGT |
| PFK1 | NM_001080244.2 | 142 | TGGAGAGTTGTGACCGCATC | TCGAAGACATAGGCAGCGTC |
| GLUT1 | XM_027537574.1 | 68 | CTGATCCTGGGTCGCTTCAT | ACGTACATGGGCACAAAACCA |
| ATPase8 | MH576694.1 | 129 | CACAATCCAGAACTGACACCAACAA | CGATAAGGGTTACGAGAGGGAGAC |

| | | | | |
|---|---|---|---|---|
| ATP5F1B | XM_027542252.1 | 152 | TGCTTTATTGGGCAGAATCC | GATCCGTCAAGTCATCAGCA |
| SDHA | XM_027520125.1 | 219 | GGGAGGACTTCAAGGAGAGG | CTCCTCAGTAGGAGCGGATG |
| SDHD | XM_015460049.1 | 113 | GTCCTATGGTGCTGGATGCT | GTTGATGTTCATGGCACAGG |
| CytC | XR_003038028.1 | 163 | CCAGGTAGCCAAGGATGTGT | CTTTCGGCTCTTGAGGACTG |
| JAK2 | XM_015464499.1 | 124 | CTAGCAGTTCGAGTTGATCA | AACAAAAGTCCACAGCAATG |
| SIRT3 | XM_010800731.3 | 152 | AGCTCATGGATCCCTTGCCT | GCTCCCCGAAGAACACGATG |
| SOD2 | NM_201527.2 | 133 | GGGAGAATGTAACTGCACGA | ACAACAGAGCAGCGTACTGG |
| VEGF | NM_174216.2 | 162 | TTCCAGGAGTACCCAGATGAGA | CTGGCTTTGGTGAGGTTTGAT |
| MAPK1 | NM_175793.2 | 169 | CAGCAACGACCACATCTGCT | GTCTGGATCTGCAACACGGG |
| GADPH | NM_001034034.2 | 185 | CCCAGAATATCATCCCTGCT | CTGCTTCACCACCTTCTTGA |

**ABV:** P21: cyclin-dependent kinase inhibitor 1; P27: cyclin-dependent kinase inhibitor 1B; GSK-3A/B: glycogen synthase kinase 3 (GSK-3) alpha and beta; GDF9: growth Differentiation Factor 9; BMP15: bone Morphogenetic Protein 15; MFN1: mitofusin-1; MFN2: mitofusin-2; EZH1: enhancer of zeste homolog 1; EZH2: enhancer of zeste homolog 2; SUZ12: suppressor of zeste 12 homolog; EED: embryonic ectoderm development; G9A: euchromatic histone lysine methyltransferase 2 (EHMT2); SETDB1: SET domain bifurcated histone lysine methyltransferase 1; SUV39H2: suppressor of variegation 3-9 homolog 2; PI3K: phosphoinositide 3-kinases; AKT: Protein kinase B; mTOR: mammalian target of rapamycin; OGG: 8-oxoguanine DNA glycosylase-1; PFK1: phosphofructokinase 1; GLUT1: glucose transporter-1; ATPase8: mitochondrial ATPase subunit 8; ATP5F1B: ATP synthase F1 subunit beta; SDHA: succinate dehydrogenase complex, subunit A; SDHD: succinate dehydrogenase subunit D; CytC: cytochrome C; JAK2: Janus kinase 2; SIRT3: sirtuin 3; SOD2: superoxide dismutase 2; VEGF: vascular endothelial growth factor; MAPK1: mitogen-activated protein kinase 1; GAPDH: glyceraldehyde-3-phosphate dehydrogenase.

**Table S2:** List of Antibodies used for immunofluorescence analysis

| Antibodies | Company | Catalogue code | Dilution |
|---|---|---|---|
| Anti-PI3K | Santa Cruz | Sc-374534 | 1:100 |
| Anti-pAkt (Ser473) | Cell Signaling | 4060 | 1:200 |
| Anti-pmTOR (S2448) | Abcam | ab84400 | 1:200 |
| Anti-LC3B | Abcam | ab51520 | 1:1000 |
| Anti-Beclin-1 | Santa Cruz | Sc-48341 | 1:200 |
| Anti-8-OxoG Lesion | Santa Cruz | Sc-130914 | 1:100 |
| Alexa Fluor-568 anti-mouse | Invitrogen | A10037 | 1:500 |
| Alexa Fluor-488 anti-rabbit | Thermo Fisher | A21206 | 1:500 |

**ABV:** pAKT: phosphorylated AKT; pmTOR: phosphorylated mTOR; LC3B (MAP1LC3B): Microtubule associated protein 1 light chain 3 beta; Beclin-1: Autophagy-related gene 6; 8-OxoG: 8-Oxoguanine.
